# Comparative Study of the Structural Characteristics and Bioactivity of Polysaccharides Extracted from *Aspidopterys obcordata* Hemsl. Using Different Solvents

**DOI:** 10.3390/molecules28247977

**Published:** 2023-12-06

**Authors:** Jia-Rui Yue, Jian-Mei Lu, Qing-Fei Fan, Peng Sun, Yang-Jian Li, Shi-Lin Zhou, Xin-Yue Wang, Jun-Mei Niu, You-Kai Xu, Jing Zhou

**Affiliations:** 1 School of Pharmaceutical Science & Yunnan Key Laboratory of Pharmacology for Natural Products, Kunming Medical University, Kunming 650500, China; 2 CAS Key Laboratory of Tropical Plant Resources and Sustainable Use, Xishuangbanna Tropical Botanical Garden, Chinese Academy of Sciences, Menglun 666303, China; 3 Dehong Vocational College, Mangshi 678400, China; 4 The Center for Gardening and Horticulture, Xishuangbanna Tropical Botanical Garden, Chinese Academy of Sciences, Menglun 666303, China; 5 College of Science, Yunnan Agricultural University, Kunming 650201, China

**Keywords:** *Aspidopterys obcordata* Hemsl., polysaccharides, extraction, antioxidant activity, oxalate crystallization

## Abstract

The polysaccharides extracted from *Aspidopterys obcordata* are thought to have anti-urolithiasis activity in Drosophila kidney stones. This study aimed to assess the effects of different extraction solvents on the yield, chemical composition, and bioactivity of polysaccharides from *A. obcordata*. *A. obcordata* polysaccharides were extracted by using four solutions: hot water, HCl solution, NaOH solution, and 0.1 M NaCl. The results revealed that the extraction solvents significantly influenced the extraction yields, molecular weight distribution, monosaccharide compositions, preliminary structural characteristics, and microstructures of polysaccharides. The NaOH solution’s extraction yield was significantly higher than the other extraction methods. Vitro antioxidant activity assays revealed that the NaOH solution extracted exhibited superior scavenging abilities towards DPPH and ABTS radicals and higher FRAP values than other polysaccharides. The vitro assays conducted for calcium oxalate crystallization demonstrated that four polysaccharides exhibited inhibitory effects on the nucleation and aggregation of calcium oxalate crystals, impeded calcium oxalate monohydrate growth, and induced calcium oxalate dihydrate formation. The NaOH solution extracted exhibited the most pronounced inhibition of calcium oxalate crystal nucleation, while the hot water extracted demonstrated the most significant suppression of calcium oxalate crystal aggregation. Therefore, it can be inferred that polysaccharides extracted with NaOH solution exhibited significant potential as a viable approach for extracting polysaccharides from stems due to their superior yield and the remarkable bioactivity of the resulting products.

## 1. Introduction

*Aspidopterys obcordata* Hemsl. (Malpighiaceae) is distributed in Hainan Province and southern Yunnan Province, China [1]. *A. obcordata* is a significant component in some ancient prescriptions of Traditional Dai Medicine. In the Dai ethnic region of China, *A. obcordata* vine (Hei Gai Guan) has been traditionally employed as a medicinal remedy for managing urinary tract stone-related ailments [2]. It is also consumed as an herbal infusion to proactively mitigate the occurrence of kidney stones. Previous studies indicated that the vine contained many bioactive constituents, including tannins, sterols, phenolic acids, polysaccharides, triterpenes, and flavonoids, and the constituents contributed to anti-inflammation and diuresis [3]. Furthermore, the vine was historically used to treat diverse ailments including urinary tract infections and stones, cystitis, rheumatism, postpartum weakness, and digestive disorders in children [4,5,6,7].

Polysaccharides play a pivotal role as the fundamental material basis for the distinctive therapeutic efficacy of Traditional Chinese Medicine [8]. Compared to synthetic drugs, plant polysaccharides exhibit favorable effects, minimal toxic side effects, and enhanced safety profiles, rendering them a promising alternative to synthetic drugs [9]. Previous studies have demonstrated that plant polysaccharide components exhibit a diverse array of biological activities, including but not limited to antitumor, antioxidant, anticoagulant, antidiabetic, antiviral, hypolipidemic, and immunomodulatory effects [10]. The presence of oxalate and calcium oxalate (CaOx) crystals in patients with kidney stones triggers the generation of free radicals within renal epithelial cells, leading to oxidative stress that ultimately culminates in the formation of kidney stones [11]. Previous studies have demonstrated the considerable therapeutic potential of antioxidants and free-radical scavengers in mitigating the occurrence and recurrence of urinary stones, while also offering enhanced renal protection [12]. The polysaccharides derived from plants not only play a role in mitigating cellular oxidative damage by scavenging free radicals [13], but also demonstrate inhibitory effects on the nucleation, aggregation, and growth of crystals [14]. Previous research conducted in our laboratory has demonstrated *A. obcordata* fructan, a polysaccharide extracted from this plant, had a significant impact on the size of calcium oxalate crystals in *Drosophila* Malpighian tubules [15]. The aforementioned outcome was achieved by impeding the pace of crystal formation and the development of large calcium oxalate crystals, as well as inducing the formation of calcium oxalate dihydrate. However, there is limited information on the extraction of polysaccharides from *A. obcordata*.

The extraction process represents the initial and pivotal stage in characterizing and harnessing bioactive polysaccharides from materials [16]. Hot water extraction is a widely employed conventional technique for the preparation and extraction of polysaccharides in laboratory and industrial settings, owing to its inherent advantages such as cost-effectiveness, ease of implementation, safety measures, and eco-friendliness [17,18]. Apart from hot water extraction, saline, acidic, and diluted alkaline solutions are widely employed for the extraction of polysaccharides. The choice of extraction solvent significantly impacts the yield, physicochemical properties, and bioactivity of natural polysaccharides [19,20,21,22]. Previous studies have revealed considerable heterogeneity in the physicochemical and bioactive properties of polysaccharides obtained through diverse extraction methods. Gao et al. [23] conducted a comparative analysis of the structural characteristics of *Lonicera japonica* polysaccharides extracted using seven different methods, revealing that the choice of extraction techniques significantly influences the yield, molecular weight, monosaccharide composition, and levels of neutral sugar, uronic acid, fucose, and sulfate in the polysaccharide. To obtain sulfated polysaccharides from *Ulva intestinalis*, Peasura et al. [24] used a series of extraction methods, including distilled water, 0.1 M NaCl, and 0.1 M HCl at 80 °C, with varying extraction times. They found that the antioxidant activity was effectively enhanced by solvent extraction due to the distinctive molecular weight and chemical composition of sulfated polysaccharides. Zhao et al. [25] used four distinct methodologies to extract polysaccharides from *Dioscorea opposita*. According to their analysis, the acid extraction technique has been determined to be a highly effective method for extracting bioactive polysaccharides from *D. opposita*. However, previous investigations on *A. obcordata* have primarily focused on the isolation, extraction, and identification of their chemical constituents, including triterpenoids, sterols, tannins, and other bioactive compounds. To date, there has been a limited comprehensive evaluation of the yield, physicochemical properties, and functional characteristics of *A. obcordata* polysaccharides obtained through various solvent extraction methods.

Therefore, in the present study, we aim to investigate the impact of four different extraction solvents including hot water, acid solution, alkali solution, and NaCl solution on the extraction rate, chemical composition, physicochemical properties, antioxidant activities, and inhibitory crystallization abilities of *A. obcordata* polysaccharides (AOPs). The in vitro antioxidant activities of four polysaccharides were compared by conducting assays on DPPH, hydroxyl, and ABTS radicals. Additionally, the inhibitory effects of the four polysaccharides on both nucleation and aggregation rates of calcium oxalate crystals were investigated via crystallization kinetic tests. The results reached from this study hold significant implications in the selection of an appropriate solvent for the efficient extraction of polysaccharides from *A. obcordata*.

## 2. Results and Discussion

### 2.1. Extraction Yields and Chemical Composition of AOPs

The polysaccharides obtained from four different extraction solvents, including hot water, acid solution, alkali solution, and sodium chloride solution were named Hw-AOPs, Ac-AOPs, Al-AOPs, and Na-AOPs, respectively. The extraction yields and physicochemical characteristics of the polysaccharides obtained from *A. obcordata* using four different methods are shown in Table 1. The results indicated that the extraction method significantly impacted both the extraction rate and physicochemical properties. The yields of AOPs varied depending on the extraction solvent, ranging from 1.66% to 3.32%. Among those AOPs, Al-AOPs exhibited the highest yield (3.32%), followed by Hw-AOPs (2.06%), Na-AOPs (2.03%), and Ac-AOPs (1.66%). This finding was consistent with the results reported by Dou et al. [26], who observed the highest yield through alkali extraction (ALE) and the lowest yield through acid extraction (ACE). Compared with hot water extraction (HWE), alkali extraction not only broke the glycoleptide bonds of glycoproteins, and strengthened the cleavage of hydrogen bonds, but also hydrolyzed alkaline and acidic polysaccharides, transforming insoluble cell wall polysaccharides into soluble polysaccharides. This process induces polysaccharide degradation and diffusion while enhancing solubility [20,24,27]. However, alkaline extraction also caused the extraction of proteins and minerals that are closely associated with polysaccharides [24]. The extraction rates of NaCl solution and hot water were nearly identical, likely due to their comparable abilities to rupture cell walls. The low yield of polysaccharides obtained by acid extraction may be attributed to the acidic conditions that cleave glycosidic bonds in polysaccharides, thereby releasing some free sugars [28]. The free sugars may not be precipitated by ethanol, leading to reduced extraction efficiency [29].

The Na-AOPs extracted using a NaCl solution exhibited the highest total sugar content (61.56%), whereas the Ac-AOPs extracted through an acidic solution demonstrated the lowest content (35.42%). The acid extraction method showed the lowest total sugar content, primarily due to the susceptibility of plant polysaccharides to partial degradation under acidic conditions [30]. The total protein contents of the four AOPs were found to be relatively low. The slightly higher protein content of Al-AOPs may be attributed to the hydrolysis of protein amide bonds by alkaline solvents, which is consistent with previous findings in blackberry fruit, *Hericium erinaceus*, and *Fructus Mori* polysaccharides extracted using an alkaline solution [20,26,31]. The Ac-AOPs exhibited the highest uronic acid content (64.31%), followed by Na-AOPs (27.26%), Al-AOPs (24.08%), and Hw-AOPs (23.14%), which indicated that the four AOPs were acidic.

### 2.2. Structure Characterizations

#### 2.2.1. Molecular Weight of AOPs

**Figure 1:**
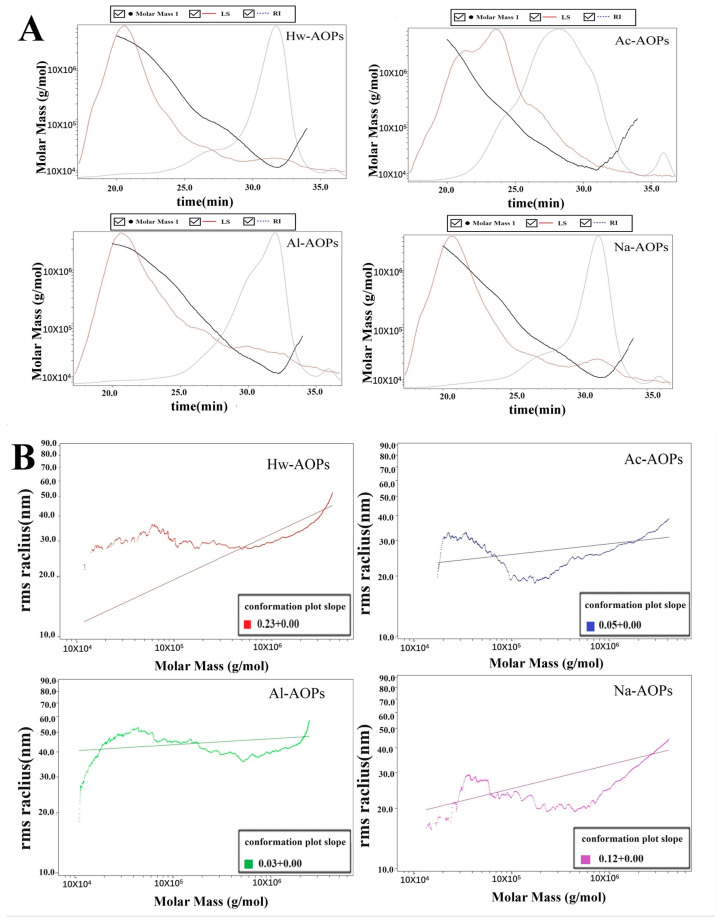
(**A**) Absolute molecular weight analysis chart of AOPs procured through different extraction methods; (**B**) molecular configuration analysis.

The biological activity of polysaccharides is intricately linked to their molecular weight, which can be significantly influenced by the extraction solvents and methods employed [32]. Table 2 and Figure 1 show that the molecular weight (Mw) distributions of the four polysaccharides were different. The molecular weight of Hw-AOPs was the highest (107.103 kDa), which may be attributed to the aggregation of polysaccharides under hot water solutions [22,33]. The lower molecular weight of Ac-AOPs and Al-AOPs was consistent with previous findings, suggesting that the acid and alkali extractions effectively disrupted the glycosidic linkages, leading to a reduction in molecular weight [33]. Similarly, Na-AOPs obtained by saline extraction showed the lowest molecular weight.

#### 2.2.2. Monosaccharide Composition

The monosaccharide compositions of four AOPs, extracted using different extraction methods, were presented in Table 3. The fucose, rhamnose, arabinose, galactose, glucose, fructose, galacturonic acid, and glucuronic acid existed with different molar ratios in Hw-AOPs (0.51%, 2.49%, 3.72%, 5.60%, 3.37%, 73.43%, 9.59%, 1.29%), Al-AOPs (0.54%, 2.03%, 4.25%, 4.61%, 2.74%, 75.01%, 9.70%, 1.13%), and Na-AOPs (0.49%, 2.00%, 2.61%, 3.27%, 1.66%, 76.32%, 12.73%, 0.91%). However, the Ac-AOPs contained fucose, rhamnose, arabinose, galactose, glucose, xylose, galacturonic acid, and glucuronic acid (3.04%, 12.50%, 14.95%, 14.53%, 3.00%, 6.11%, 42.43%, 3.44%). The presence of galacturonic acid and glucuronic acid was observed in all four AOPs, with a higher galacturonic acid in the Ac-AOPs (42.43%), indicating that these four AOPs were acidic heteropolysaccharides. The present finding is in line with previous studies, which suggest that the extraction solvent had significant impacts on the types and ratios of monosaccharides [34].

#### 2.2.3. UV Spectroscopic Analysis of AOPs

**Figure 2:**
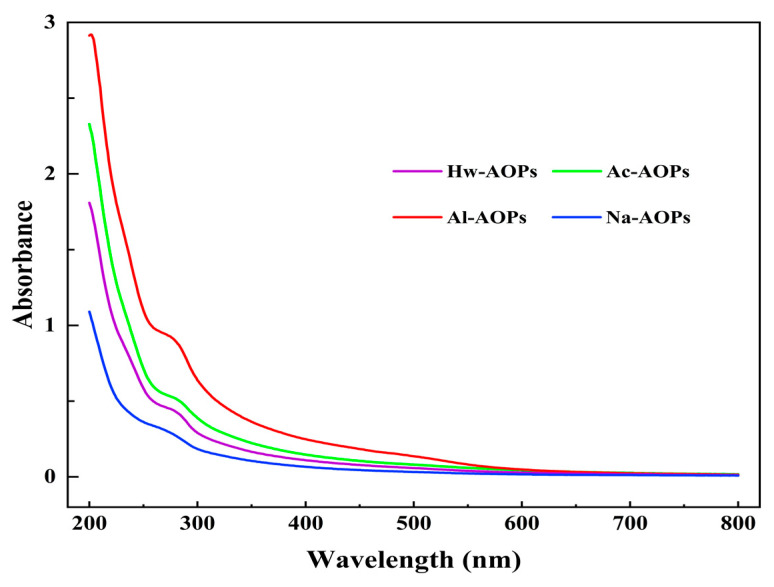
The UV spectra of four polysaccharides were acquired through different extraction methods.

The UV spectra of AOPs are shown in Figure 2. The protein absorption peaks at 280 nm were detected for both Al-AOPs and Ac-AOPs, indicating the potential presence of protein and nucleic acid within these two polysaccharides. The spectral curves of Hw-AOPs and Na-AOPs exhibited a relatively smooth profile, suggesting that a minimal or insignificant protein and nucleic acid content existed. This outcome was in line with the findings from the chemical analysis.

#### 2.2.4. FT-IR Spectroscopy of AOPs

**Figure 3:**
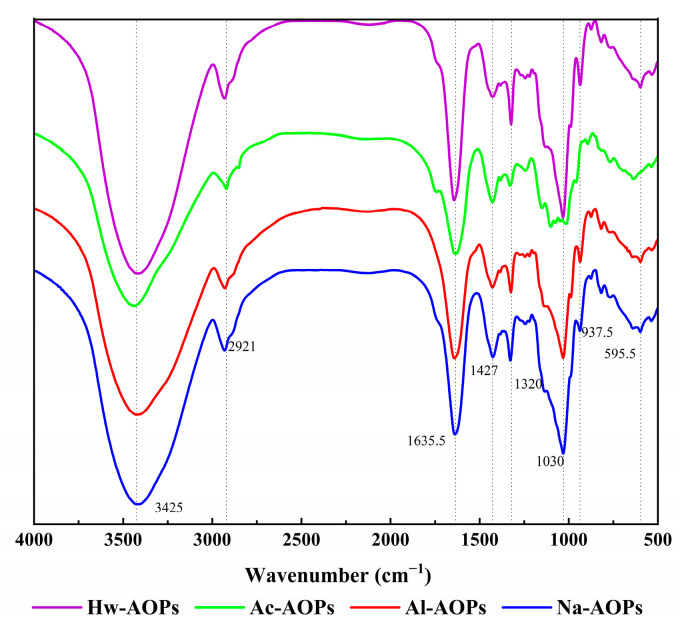
FT−IR spectra of four polysaccharides extracted by different methods.

FT-IR spectroscopy was used to further investigate the structural characteristics of four AOPs. The structures of the four AOPs exhibited a high similarity, as illustrated in Figure 3. The characteristic peaks of polysaccharides were observed at frequencies around 3400, 2930, 1640, 1425, and 1030 cm^−1^ in the AOPs samples [35]. The strong and broad absorption band observed at approximately 3400 cm^−1^ was indicative of the presence of O-H stretching vibrations, which was attributed to both intramolecular and intermolecular interactions within the polysaccharide chains [36]. The weaker absorption peak at 2930 cm^−1^ was the stretching vibration of the sugar C-H [37]. The absorption peaks at 1640 cm^−1^ and 1420 cm^−1^ were assigned to asymmetric and symmetric C=O stretching vibrations, further indicating that these AOPs are acidic [38,39]. Meanwhile, there was no significant adsorption at 1730 cm^−1^, indicating the uronic acids in the four AOPs were non-esterified [40,41]. The absorption peaks within the 1200–950 cm^−1^ range can be classified as either C-O-H variable angle vibrational absorption peaks or C-O-C stretching vibrational absorption peaks in the pyranose ring [42].

#### 2.2.5. SEM Analysis AOPs-

**Figure 4:**
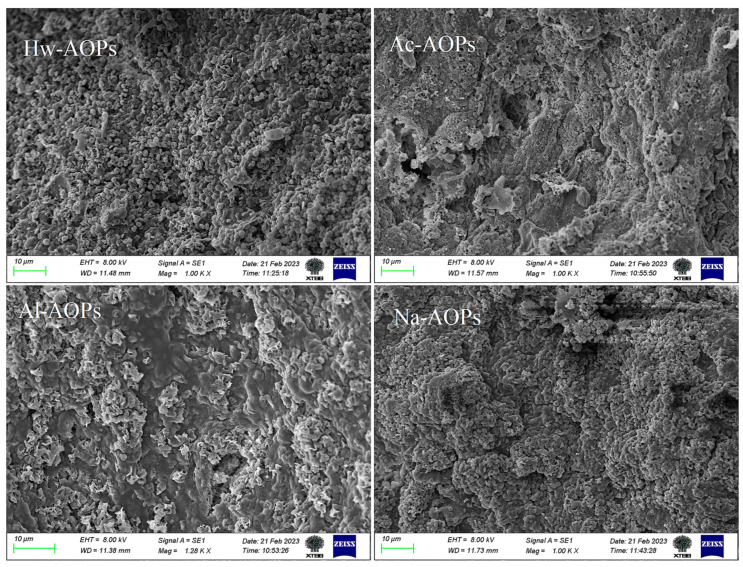
SEM photographs of AOPs obtained by different extraction methods (×1000).

Scanning electron microscopy (SEM) is a valuable tool for polysaccharide structural analysis, as it enables the observation of surface morphological features and structural differences in samples [43]. Therefore, scanning electron microscopy (SEM) is widely utilized for the examination of the morphology and microstructural features of polysaccharides. As shown in Figure 4, the surface morphology of AOPs extracted by different solvents showed significant differences in shape and size. The surface of polysaccharides extracted by hot water (Hw-AOPs) was rough, with more granular spheroids. Na-AOPs extracted by saline solution were similar to the Hw-AOPs, with relatively few granular spheroids. The surface of Ac-AOPs, however, exhibited a relatively loose structure with small fissures, suggesting that the addition of hydrochloric acid may disrupt the polysaccharide architecture and reduce its overall size. The surface of Al-AOPs extracted by alkali solution exhibited irregularly granular or reef-shaped morphology, characterized by a rough texture. These findings suggested that the choice of extraction solutions can significantly impact the surface morphology of polysaccharides.

### 2.3. In Vitro Antioxidant Activity of AOPs

#### 2.3.1. DPPH Radical Scavenging Assay

**Figure 5:**
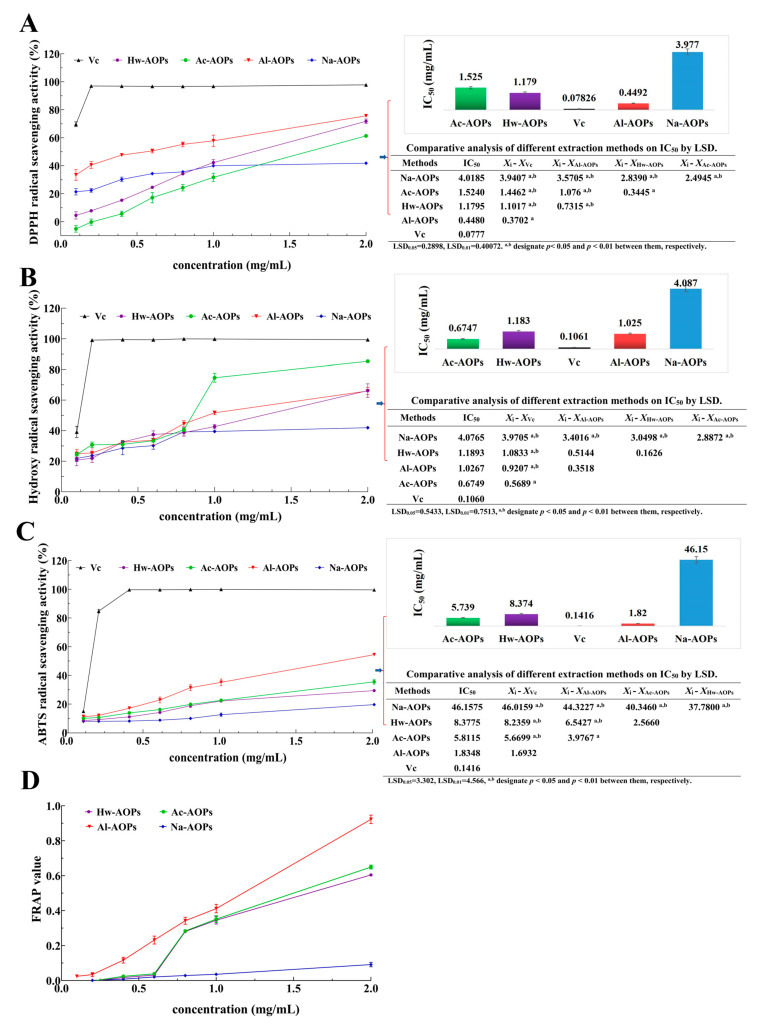
Antioxidant activities of AOPs. (**A**) DPPH radical scavenging activity; (**B**) hydroxyl radical scavenging activity; (**C**) ABTS radical scavenging activity; (**D**) FRAP assay.

The assessment models of DPPH radical scavenging are extensively employed for evaluating the antioxidant activity of natural products [44]. As depicted in Figure 5A, four samples of AOPs exhibited the ability to scavenge DPPH radicals, and this scavenging activity exhibited a positive correlation with the concentration of AOPs. At the concentration of 2 mg/mL, the DPPH radical scavenging rates of Na-AOPs, Ac-AOPs, Hw-AOPs, and Al-AOPs were found to be 41.82%, 61.30%, 71.78%, and 75.67%, respectively. The 50% inhibitory concentration (IC_50_) values of Na-AOPs, Ac-AOPs, Hw-AOPs, and Al-AOPs were calculated to be 4.02, 1.52, 1.18, and 0.45 mg/mL, respectively. The Al-AOPs exhibited the highest DPPH radical scavenging capacity among the four polysaccharide samples, potentially attributed to the alkaline environment that facilitated the solubilization of polyphenols and proteins. The DPPH radical scavenging activity of the Hw-AOPs was superior to the Ac-AOPs, consistent with previous findings reported by Chen et al. [31].

#### 2.3.2. Hydroxyl Radicals Scavenging Assay

The scavenging rate of hydroxyl radicals serves as a crucial indicator for assessing antioxidant activity. Hydroxyl radicals are considered to be one of the most potent oxidative species, capable of inflicting severe damage on a wide range of biomolecules such as DNA, proteins, and polyunsaturated fatty acids within living cells, thereby inducing cytotoxicity, mutagenicity, carcinogenicity, and other deleterious effects [45,46]. The hydroxyl radical scavenging activity was assessed based on the Fenton reaction principle, and the results are depicted in Figure 5B. The scavenging capacity of the four polysaccharide samples for free radicals increased with increasing concentrations within the range of 0.1–2.0 mg/mL. At the concentration of 2 mg/mL, the hydroxyl radical scavenging rates of Na-AOPs, Al-AOPs, Hw-AOPs, and Ac-AOPs were found to be 41.91%, 65.94%, 66.19%, and 85.29%, respectively. The IC_50_ values of Na-AOPs, Hw-AOPs, Al-AOPs, and Ac-AOPs were calculated to be about 4.07, 1.19, 1.03, and 0.67 mg/mL, respectively. The remarkable hydroxyl radical scavenging ability of Ac-AOPs may be attributed to their high uronic acid content, consistent with previous findings reported by Sun et al. [47].

#### 2.3.3. ABTS Radical Scavenging Assay

ABTS is a decolorization assay that has been utilized for the assessment of hydrophilic and lipophilic antioxidants [48]. The absorbance at 734 nm of the polysaccharide sample and the positive control may serve as an indicator for evaluating the antioxidant activity of the polysaccharide [49]. As illustrated in Figure 5C, the four AOP samples demonstrated a dose-dependent increase in ABTS radical scavenging activity within the measured range (0.1–2.0 mg/mL). At the concentration of 2 mg/mL, the scavenging abilities of Na-AOPs, Hw-AOPs, Ac-AOPs, and Al-AOPs on ABTS radicals were 19.65%, 29.38%, 35.51%, and 54.42%, respectively. The ABTS radical scavenging ability of Na-AOPs increased slowly in a dose-dependent manner, and the scavenging rate was only 16.98% at the concentration of 2 mg/mL, which was much lower than 50%. The IC_50_ values of Na-AOPs, Hw-AOPs, Ac-AOPs, and Al-AOPs were calculated to be 46.16, 8.38, 5.81, and 1.83 mg/mL, respectively. The Al-AOPs exhibited relatively robust ABTS radical scavenging activity, which was consistent with the results of DPPH radicals. The promotion of phenol and protein release in the alkaline environment may account for this phenomenon. Relevant findings have also been reported in previous studies [20,50].

#### 2.3.4. Ferric Reducing Antioxidant Power

The reduction capacity serves as a crucial indicator of the antioxidant efficacy of natural products, which involves a reduction reaction facilitated by antioxidants through electron donation to scavenge free radicals [51]. The correlation between the reducing power of antioxidants and their antioxidant activity is highly significant, thus enabling the measurement of their antioxidant properties through the magnitude of their reducing power. The FRAP assay measures the ability of antioxidants to reduce Fe^3+^-TPTZ under acidic conditions, resulting in the formation of blue Fe^2+^-TPTZ with a maximum absorption peak at 593 nm. The addition of antioxidants enhances the production of blue Fe^2+^-TPTZ. The sample solution’s Fe^2+^ concentration (μmol/L) was calculated from the standard curve using the absorbance value measured at 593 nm as the FRAP value. A higher FRAP value indicates a greater total reducing capacity of the antioxidant [52]. The FRAP values of various concentrations of AOPs are presented in Figure 5D. The FRAP values of AOPs exhibited a dose-dependent increase with a rising concentration within the range of 0.1–2 mg/mL for the sample. The FRAP values at 2 mg/mL concentrations for Na-AOPs, Hw-AOPs, Ac-AOPs, and Al-AOPs were 0.09, 0.60, 0.65, and 0.94, respectively. The FRAP value of Al-AOPs demonstrated superior activity compared to the other three AOPs, potentially attributed to the alkaline conditions facilitating the leaching of impurities possessing reducing properties, such as polyphenols, pigments, and proteins.

### 2.4. CaOx Crystallization Analysis

#### 2.4.1. Effect on Crystal Morphology

**Figure 6:**
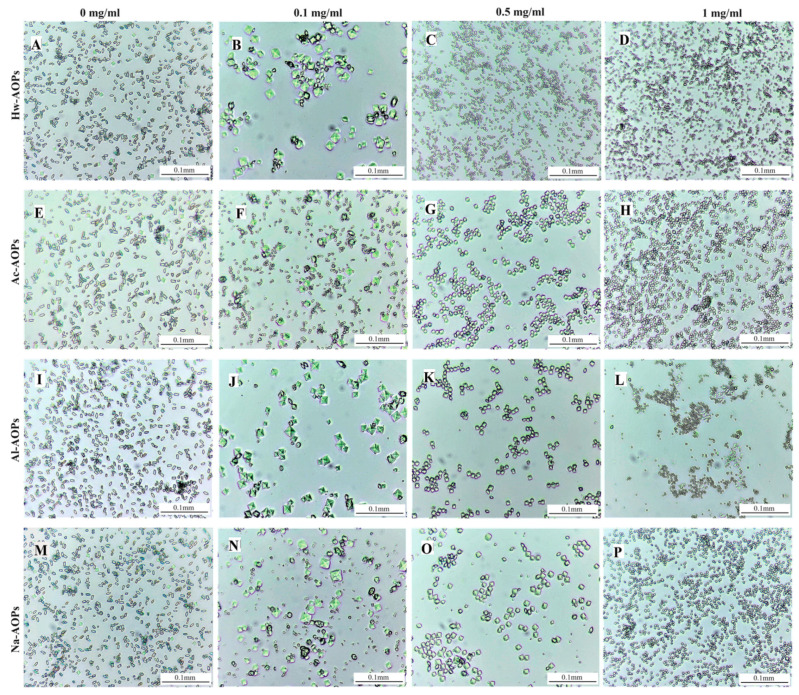
Light micrographs of CaOx crystals grown in the absence and presence of AOPs. (**A**,**E**,**I**) and (**M**) controls; (**B**) Hw-AOPs, 0.1 mg/mL; (**C**) Hw-AOPs, 0.5 mg/mL; (**D**) Hw-AOPs, 1 mg/mL; (**F**) Ac-AOPs, 0.1 mg/mL; (**G**) Ac-AOPs, 0.5 mg/mL; (**H**) Ac-AOPs, 1 mg/mL; (**J**) Al-AOPs, 0.1 mg/mL; (**K**) Al-AOPs, 0.5 mg/mL; (**L**) Al-AOPs, 1 mg/mL; (**N**) Na-AOPs, 0.1 mg/mL; (**O**) Na-AOPs, 0.5 mg/mL; (**P**) Na-AOPs, 1 mg/mL. Magnification was 200× for all panels. Ac-AOPs.

The crystallization of CaOx in the presence and absence of AOPs is illustrated in Figure 6. In the absence of AOPs, two distinct types of CaOx crystals were observed: an abundance of calcium oxalate monohydrate (COM) crystals exhibiting monoclinic prismatic shapes or irregular morphology, and a limited number of calcium oxalate monohydrate (COD) crystals displaying characteristic bipyramidal morphology (Figure 6A,E,I,M).

The addition of AOPs extracted In the reaction mixture induces modifications in the crystal structure, quantity, and dimensions. In the presence of 0.1 mg/mL AOPs, COD crystals predominantly exhibited blunt edges and corners. In the presence of Ac-AOPs, there was a significant increase in the number of COD crystals, while the number of COM crystals was significantly reduced and their morphology became thinner and rounder (Figure 6F). The crystals induced by Al-AOPs exhibited larger dimensions compared to those formed by Na-AOPs or Hw-AOPs (Figure 6B,J,N). The exclusive presence of the COD form was observed at higher concentrations of AOPs, ranging from 0.5 to 1 mg/mL. The formation of COD crystals exhibited a dose-dependent increase with rising concentrations of the extract, concomitant with a progressive reduction in crystal size. At the highest concentrations, a substantial quantity of minute crystals was generated. The presence of COD may inhibit the formation of kidney stones, as crystals with this morphology are more readily eliminated through urine compared to those composed of COM [12]. The smaller the size of COD crystals, the more easily they can be eliminated from the body through urine excretion.

#### 2.4.2. Spectrophotometric Crystallization Measurements

**Figure 7:**
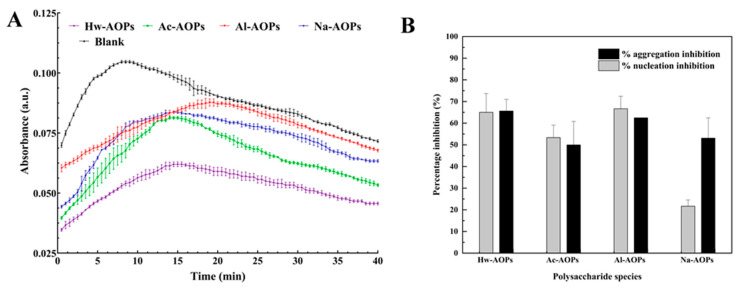
(**A**) Time-course measurements of OD_620_ in five control experiments at standard conditions (4 mmol/L CaCl_2_ and 0.5 mmol/L Na_2_O_x_). (**B**) Percentage inhibition of calcium oxalate crystal nucleation and aggregation by four AOPs. *c*(AOPs) = 0.5 mg/mL.

The effects of the four AOPs on the nucleation and aggregation of CaOx crystals are illustrated in Figure 7. The addition of the four AOPs resulted in a prolonged nucleation t_max_ of the crystals, as depicted in Figure 7 and Table 4. Additionally, there was a reduction observed in both the S_N_ and S_A_ of CaOx crystal. The nucleation inhibition rates of Hw-AOPs, Ac-AOPs, Al-AOPs, and Na-AOPs at a concentration of 0.5 mg/mL on CaOx crystallization were determined to be 65.00%, 53.33%, 66.67%, and 21.67%, respectively, while the aggregation rates of CaOx crystals were observed to be 65.60%, 49.97%, 62.48%, and 53.10%. The inhibitory effect of AOPs on CaOx crystal nucleation followed the following order: Al-AOPs > Hw-AOPs > Ac-AOPs > Na-AOPs. In terms of aggregation, Hw-AOPs showed the strongest ability to promote aggregation, followed by Al-AOPs, Na-AOPs, and Ac-AOPs.

## 3. Materials and Methods

### 3.1. Biological Materials and Chemicals

The stem of *A. obcordata* was collected in 2022 from Xishuangbanna Tropical Botanical Garden (XTBG), Chinese Academy of Science (CAS), located in Mengla County, Yunnan Province, China. The monosaccharide standards (analytical standard, chromatographic purity), sodium hydroxide, and sodium acetate trihydrate were purchased from Sigma-Aldrich Chemical Co. (St. Louis, MO, USA). The 3-phenyl phenol, sodium tetraborate, brilliant blue G, and albumin from bovine serum (BSA) were purchased from Aladdin Biochemical Technology Co., Ltd. (Shanghai, China). Iron (II) sulfate heptahydrate, ascorbic acid, pyrogallol, 2,2′-azinobis (3-ethylbenzothiazoline-6-sulphonic acid) (ABTS), potassium persulfate, 2,4,6-tris(2-pyridyl)-s-triazine (TPTZ), Tris (hydroxymethyl) aminomethane (Tris base), salicylic acid, and iron (III) chloride were procured from Meryer (Shanghai) Chemical Technology Co., Ltd. (Shanghai, China). Phenol was procured from Shanghai Macklin Biochemical Co., Ltd. (Shanghai, China). All other chemicals and reagents were chromatographic or analytical grade and obtained from local suppliers.

### 3.2. Polysaccharide Extraction and Isolation

#### 3.2.1. Pretreatment of *A. obcordate* Stem

The stem of *A. obcordata* was dried at 45 °C in an oven, pulverized as fine powder, and passed through a 40-mesh screen. The dried stem of *A. obcordata* powder (500 g) was extracted with 95% ethanol for 4 h at 70 °C for 3 times to remove soluble materials, including free sugars, amino acids, and some phenols. The residue was vacuum filtration and dried at 40 °C to obtain pretreated dry powder for further use.

#### 3.2.2. Polysaccharide Extraction

**Figure 8:**
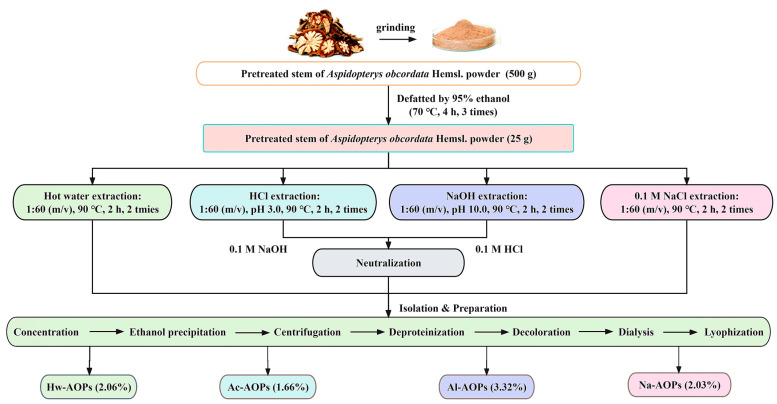
The flowchart of extraction and isolation by different methods.

The extraction solvents included deionized water, an aqueous solution of HCl (pH 3.0), an aqueous solution of NaOH (pH 10.0), and a 0.1 M aqueous solution of NaCl. The pretreated powder (25 g) was extracted 2 times for 2 h with the residue to solvent ratio of 1:60 (*w*/*v*), the temperature of extraction was controlled at 90 °C by a thermostat water bath. After extraction, the supernatant of HCl and NaOH solutions was neutralized with 0.1 M NaOH and 0.1 M HCl, and then centrifugation (5000 r/min, 5 min), respectively. The extraction solution underwent pressure reduction and was subsequently concentrated to 1/4 of its initial volume. Subsequently, anhydrous ethanol was added in a quantity four times that of the concentrated solution, followed by thorough mixing and overnight incubation at 4 °C. Then, the solution was centrifuged at 5000 r/min for 15 min, the supernatant was removed, the precipitation was collected, and the precipitation was cleaned with anhydrous ethanol and acetone three times in a turn. The precipitate was redissolved in deionized water, followed by deproteinization using trichloroacetic acid (TCA), decolorization employing D101 macroporous resin, and dialysis with tap water and distilled water for 48 h (molecular cut-off of 2000 Da). Subsequently, the solution was freeze dried and named Hw-AOPs, Ac-AOPs, Al-AOPs, and Na-AOPs samples. The procedures for detailed extraction and isolation of AOP are summarized in Figure 8.

### 3.3. Chemical Composition Analysis

The total sugar content of the polysaccharides was quantified using the phenol-sulfuric acid method as described by DuBois et al. [53]. The protein content of the polysaccharides was determined utilizing the Coomassie brilliant blue assay, in accordance with the methodology outlined by Zor et al. [54]. The quantification of uronic acid contents was performed utilizing the 3-Phenylphenol colorimetric method, following the protocol established by Blumenkrantz et al. [55].

### 3.4. Determination of Molecular Weight and Analysis of Monosaccharide Composition

The weight-average molecular weight (Mw) distribution and polydispersity (Mw/Mn) of the four AOPs fractions were determined using SEC-MALLS-RI, following the methodology proposed by Wu et al. [56]. The AOPs powder (4.0 mg) was dissolved in 4 mL of 0.1 M NaNO_3_ aqueous solution containing 0.02% NaN_3_, filtered through 0.45 μm membranes. The sample solution (100 μL) was injected and measured on a DAWN HELEOS-II laser photometer (Wyatt Technology Co., Goleta, CA, USA) equipped with two tandem columns (300 × 8 mm, Shodex OH-pak SB-805 and 803; Showa Denko K.K., Tokyo, Japan) at a flow rate of 0.6 mL/min and a column temperature of 45 °C. The concentration of fractions and the dn/dc value were simultaneously determined using a differential refractive index detector (Optilab T-rEX, Wyatt Technology Co., USA). The mobile phase consisted of a 0.1 mol/L NaNO_3_ solution containing 0.02% NaN_3_.

The monosaccharide composition of 4 AOPs was using the method proposed by Wang et al. [57]. The polysaccharide (5 mg) was hydrolyzed by adding 1 mL of trifluoroacetic acid (TFA) at a concentration of 2 mol/L, followed by incubation in an oven at 121 °C for a duration of 2 h. The sample was dried using nitrogen gas, followed by rinsing with methanol and gentle drying. The methanol rinse process was repeated 2–3 times. The remaining substance was then dissolved in deionized water and passed through a microporous filtering film with a pore size of 0.22 μm for subsequent analysis. The monosaccharide component of hydrolyzed AOPs was analyzed using a high-performance anion exchange chromatography (HPAEC) system (ICS 5000, Thermo Fisher Scientific, Waltham, MA, USA), equipped with a CarboPac PA-20 anion-exchange column (3 by 150 mm; Dionex) and a pulsed amperometric detector. The injection volume was 5 μL. The column temperature for mobile phase A (H_2_O), mobile phase B (100 mM NaOH), and mobile phase C (0.1 M NaOH, 0.2 M NaAc) was maintained at 30 °C. Gradient program, volume ratio of Solutions A, B, and C were 95:5:0 at 0 min, 85:5:10 at 26 min, 85:5:10 at 42 min, 60:0:40 at 42.1 min, 60:40:0 at 52 min, 95:5:0 at 52.1 min, and 95:5:0 at 60 min.

In order to determine the fructose content of the four AOPs, 5 mg of AOPs were subjected to hydrolysis with trifluoroacetic acid (2 M) in a sealed tube. This process was carried out at a temperature of 60 °C for a duration of 30 min. The methodologies and protocols utilized in the previous paragraph for ascertaining the composition of monosaccharides remain unchanged during subsequent procedures and determinations.

### 3.5. UV Spectroscopy Analysis and FT-IR Spectroscopic Analyses

The polysaccharide samples were accurately weighed, dissolved in deionized water, and prepared into a 1 mg/mL solution. The solution was centrifuged at 5000 r/min for 10 min after complete dissolution. The resulting supernatant was analyzed using a UV-Vis spectrophotometer (Thermofisher, BIONANO, Waltham, MA, USA) in the wavelength range of 190–800 nm with distilled water as a reference and following the same centrifugation procedure.

The infrared absorption spectra of polysaccharide samples were determined by the KBr tablet press method of Wu et al. [58]. An amount of 2 mg of sample and 200 mg of pre-dried spectrally pure KBr powder were weighed and ground well in a dry agate mortar, pressed with a tablet press, and the samples were scanned by FTIR spectrometer (Thermo Fisher, NICOLET iS10, USA) in the range of 450–4000 cm^−1^ using KBr as control.

### 3.6. Scanning Electron Microscopy (SEM)

The surface morphology of each sample was examined using a scanning electron microscope (EVO LS10, Carl Zeiss, Shanghai, China) at an acceleration voltage of 8 kV.

### 3.7. In Vitro Antioxidant Activity

#### 3.7.1. DPPH Radical Scavenging Assay

The DPPH radical scavenging assay of the AOPs was measured by the method from Andréa et al. [59] with slight modifications. An amount of 100 μL of polysaccharide sample solutions with varying concentrations (0.1, 0.2, 0.4, 0.6, 0.8, 1.0, 2.0 mg/mL) were mixed with 100 μL DPPH solution (0.4 mmol/L in methanol). The mixture was incubated in darkness at room temperature for 30 min, followed by quantification of absorbance at 517 nm using a Varioskan Flash Multimode Reader (ThermoFisher, USA). Vitamin C (Vc) was employed as a positive control throughout the experiment. The formula for calculation was as follows:

DPPH radical scavenging rate (%) = [1 − (As − Ab)/Ao] × 100%

where As denotes the absorbance value of the sample with DPPH solution, Ao denotes the absorbance value of the mixture without the sample (deionized water was utilized in lieu of the polysaccharide solution), and Ab denotes the absorbance value of the sample without DPPH solution.

#### 3.7.2. Hydroxyl Radical Scavenging Assay

The hydroxyl radical scavenging activity of AOPs was confirmed using the method of Smirnoff and Cumbes [60] with minor adaptations. An amount of 100 μL of polysaccharide sample solutions at different concentrations (0.1, 0.2, 0.4, 0.6, 0.8, 1.0, 2.0 mg/mL) was mixed with FeSO_4_ solution (9 mmol/L, 15 μL), salicylic acid solution (9 mmol/L, 15 μL), and H_2_O_2_ solution (9.8 mmol/L, 15 μL), and the reaction was carried out in a water bath at 37 °C for 30 min. The absorbance values were measured at 510 nm. Vc was used as a positive control. The calculation formula was as follows:

Hydroxyl radical scavenging rate (%) = [1 − (As − Ab)/Ao] × 100%

where As is the absorbance value of the polysaccharide sample group; Ao is the absorbance value when replacing polysaccharide sample solution with an equal amount of deionized water; Ab is the absorbance value when replacing H_2_O_2_ solution with an equal amount of deionized water.

#### 3.7.3. ABTS Radical Scavenging Assay

The ABTS radical scavenging activities of AOPs were determined using the method of Re et al. [61] with slight modifications. In brief, the solution of ABTS radical cation was produced through the interaction of 7 mmol/L ABTS solution and 2.45 mmol/L K_2_S_2_O_8_ in the dark at room temperature for 12–16 h. Then, the mixture was diluted with deionized water to an absorbance value of 0.7 ± 0.02 at 734 nm. An amount of 10 μL of AOPs solutions with different concentrations (0.1, 0.2, 0.4, 0.6, 0.8, 1.0, 2.0 mg/mL) was mixed with 190 μL of prepared ABTS solution and shaken well. Then, the mixture was reacted in the dark for 20 min, and the absorbance was determined at 734 nm. Vc was used as a positive control. The calculation formula was as follows:

ABTS radical scavenging rate (%) = [1 − (As − Ab)/Ao] × 100%

where As represents the absorbance value of polysaccharide solution, Ao represents the absorbance value of deionized water instead of polysaccharide, and Ab represents the absorbance value of deionized water instead of ABTS solution.

#### 3.7.4. Ferric-Reducing Antioxidant Capacity

The FRAP value of 4 AOPs was measured by the method Jia et al. [62], with minor adaptations.

The TPTZ working solution is prepared by thoroughly mixing 25 mL of 300 mmol/L acetate buffer solution (pH 3.6), 2.5 mL of 10 mmol/L TPTZ solution, and 2.5 mL of 20 mmol/L FeCl_3_ solution.

Standard curve: 10 μL of FeSO_4_ solution with series concentrations (0, 125, 250, 500, 750, 1000, 1500, 2000 μmol/L) was mixed with 180 μL TPTZ working solution, thoroughly mixed while avoiding light at 37 °C for 30 min, measuring the absorbance at 593 nm. The standard curve was plotted with the concentration of FeSO_4_ solution as the horizontal coordinate and the absorbance value as the vertical coordinate.

Sample determination: 10 μL of polysaccharide samples at different concentrations (0.1, 0.2, 0.4, 0.6, 0.8, 1.0, 2.0 mg/mL) were taken and mixed with 180 μL TPTZ working solution, and distilled water was used as blank control instead of polysaccharide sample solution, and the absorbance values were measured at 593 nm after 30 min reaction at 37 °C in the dark. The FRAP value of the sample solution was determined by obtaining its corresponding FeSO_4_ concentration (μmol/L) from the standard curve.

### 3.8. CaOx Crystallization Analysis

#### 3.8.1. Stock Solutions

Fresh stock solutions were prepared as follows: Solution A 12 mM calcium chloride (CaCl_2_) in 300 mM sodium chloride (NaCl) and 15 mmol/L sodium acetate (NaAc), pH 5.7; Solution B 1.5 mM sodium oxalate (Na_2_C_2_O_4_), pH 5.7 [63].

#### 3.8.2. Image Analysis of Crystal Morphology

The formation of calcium oxalate (CaOx) crystals was observed under light microscopy in the presence and absence of AOPs; 0.4 mL aliquots of CaCl_2_ solutions and varying concentrations of AOPs were dispensed into wells of a 48-well plate. The sodium oxalate solution (0.4 mL) was added to each well to achieve final concentrations of 4 mmol calcium and 0.5 mmol oxalate. The concentrations of the extracts used ranged from 0.1 to 1 mg/mL, with a sample containing 0.4 mL distilled water instead of AOPs serving as the reference control system. The plates were incubated in a water bath and maintained at 37 °C for 45 min. Subsequently, the plate was observed with an inverted microscope (Olympus Corporation, Tokyo, Japan).

#### 3.8.3. CaOx Crystallization Assay

The crystallization of calcium oxalate was investigated by monitoring the time-course changes in optical density, both in the presence and absence of AOPs following the experimental protocol described by De Bellis et al. [63]. Solutions A and B were maintained at 37 °C. The following components were sequentially added to a 3 mL glass cuvette: 1 mL of Solution A, 1 mL of AOPs (0.5 mg/mL), and 1 mL of Solution B, resulting in final assay concentrations of 5.0 mM calcium and 0.5 mM oxalate. The turbidity of the mix was quantified by measuring its absorbance at 620 nm. The measurement of absorbances at 30 s intervals over a period of 40 min was utilized to ascertain the rates at which crystals were formed. The effect of AOPs on CaOx crystallization was assessed using distilled water as a blank.

The crystallization process was characterized by the following three parameters [64]: first, crystal S_N_, which was the maximum slope of increase in OD_620_ over time and primarily signifies the maximum rate of formation of fresh particles, thereby indicating crystal nucleation; second, S_A_, the maximum slope of OD_620_ over the decrease in time reflect the degree of particle reduction caused by crystal aggregation [65]; and third, maximum time (t_max_) where neither nucleation nor growth can occur [66].

The percentage inhibition was calculated as follows: [1 − (S_NT_/S_NC_)] × 100 for the rate of nucleation and [1 − (S_AT_/S_AC_)] × 100 for the rate of aggregation, where S_NC_ and S_AC_ stand for the slopes of the blank, S_NT_ and S_AT_ stands for the slopes in the presence of the AOPs.

### 3.9. Statistical Analysis

All data were shown as the mean ± standard deviation (SD). The statistical analysis was implemented by SPSS software (Version 27.0, Chicago, IL, USA). Multiple comparisons of means were carried out by the least significant difference (LSD) test. Analysis of variance (ANOVA) was implemented and the fitness of the polynomial model equation was expressed as the coefficient of determination *R*^2^. Values of *p* < 0.05 were considered to be statistically significant. The figures were generated using Origin 2022 software (Origin Lab Corp., Northampton, MA, USA).

## 4. Conclusions

In this study, hot water, HCl aqueous solution, NaOH aqueous solution, and NaCl aqueous solution were used to extract polysaccharides (Hw-AOPs, Ac-AOPs, Al-AOPs, and Na-AOPs) from the stem of *A. obcordata*. The results showed that the extraction solvent significantly impacted the physicochemical properties, structure, surface morphology, and antioxidant activity of AOPs. Al-AOPs extracted with alkali solution showed the highest extraction efficiency and higher scavenging abilities on DPPH and ABTS radicals, as well as higher FRAP value. The Al-AOPs exhibited the most pronounced inhibition of calcium oxalate crystal nucleation, while the Hw-AOPs demonstrated the most significant suppression of calcium oxalate crystal aggregation. Thus, our study suggests that the utilization of alkaline solution extraction was a viable method for producing polysaccharides from the stem of *A. obcordata*.

## Figures and Tables

**Table 1:** The extraction yield and chemical composition of four AOPs were procured through different extraction methods.

Item	Sample			
	Hw-AOPs	Ac-AOPs	Al-AOPs	Na-AOPs
Yield (%)	2.06	1.66	3.32	2.03
Total sugar (%)	56.35 ± 0.63 ^b^	35.42 ± 0.59 ^d^	51.99 ± 2.19 ^c^	61.56 ± 0.88 ^a^
Protein (%)	nd	0.16 ± 0.12	0.23 ± 0.17	nd
Uronic acid (%)	23.14 ± 2.02 ^b^	64.31 ± 2.82 ^a^	24.08 ± 3.23 ^b^	27.26 ± 1.04 ^b^

Hw-AOPs obtained through hot water; Ac-AOPs obtained through a HCL solution; Al-AOPs obtained through a NaOH solution; Na-AOPs obtained through a NaCl solution. Values are expressed as mean ± standard deviation (*n* = 3). Means within a row with different superscripts differ significantly (*p* < 0.05). nd: not detected.

**Table 2:** Molecular weight (Mw) distribution of AOPs procured through different extraction methods.

Samples	Mw (kDa)	Mn (kDa)	Mw/Mn
Hw-AOPs	107.103	19.112	5.604
Ac-AOPs	78.715	26.166	3.008
Al-AOPs	81.037	19.635	4.127
Na-AOPs	77.534	16.195	4.788

Hw-AOPs obtained through hot water; Ac-AOPs obtained through an HCL solution; Al-AOPs obtained through a NaOH solution; Na-AOPs obtained through a NaCl solution.

**Table 3:** Monosaccharide composition of AOPs (molar ratio, %).

Samples	Hw-AOPs	Ac-AOPs	Al-AOPs	Na-AOPs
Fucose	0.51	3.04	0.54	0.49
Rhamnose	2.49	12.50	2.03	2.00
Arabinose	3.72	14.95	4.25	2.61
Galactose	5.60	14.53	4.61	3.27
Glucose	3.37	3.00	2.74	1.66
Xylose	nd	6.11	nd	nd
Fructose	73.43	nd	75.01	76.32
Galacturonic acid	9.59	42.43	9.70	12.73
Glucuronic acid	1.29	3.44	1.13	0.91

Hw-AOPs obtained through hot water; Ac-AOPs obtained through an HCL solution; Al-AOPs obtained through a NaOH solution; Na-AOPs obtained through a NaCl solution. nd: not detected.

**Table 4:** Effects of AOPs on calcium oxalate crystallization.

	Blank	Hw-AOPs	Ac-AOPs	Al-AOPs	Na-AOPs
t_max_ (min)	8.67 ± 0.29 ^d^	15.67 ± 0.76 ^b^	14.33 ± 0.29 ^c^	19.17 ± 1.25 ^a^	13.83 ± 0.58 ^c^
S_N_ (×10^−3^/min)	10.00 ± 0 ^a^	3.50 ± 0.87 ^d^	4.67 ± 0.58 ^c^	3.33 ± 0.58 ^d^	7.83 ± 0.28 ^b^
S_A_ (×10^−3^/min)	5.33 ± 0.56 ^a^	1.80 ± 0.29 ^d^	2.67 ± 0.58 ^bc^	2.00 ± 0 ^cd^	2.50 ± 0.5 0^b^

Note: t_max_ (min): the moment at which maximum absorbance; S_N_: rate of crystal nucleation; S_A_: rate of crystal aggregation. The values are presented as mean ± SD (n = 3). Means within a row with different superscripts differ significantly (*p* < 0.05). *c*(AOPs) = 0.5 mg/mL.

## Data Availability

Data are contained within the article.
